# *Strobilanthes crispus* elicits anti-tumor immunogenicity in *in vitro* and *in vivo* metastatic breast carcinoma

**DOI:** 10.1371/journal.pone.0271203

**Published:** 2022-08-16

**Authors:** Yusha’u Shu’aibu Baraya, Chee Lee Wee, Zulkarnain Mustapha, Kah Keng Wong, Nik Soriani Yaacob

**Affiliations:** 1 Department of Chemical Pathology, School of Medical Sciences, Universiti Sains Malaysia, Kubang Kerian, Kelantan, Malaysia; 2 Faculty of Veterinary Medicine, Department of Veterinary Pathology, Usmanu Danfodiyo University, Sokoto, Nigeria; 3 Department of Immunology, School of Medical Sciences, Universiti Sains Malaysia, Kubang Kerian, Kelantan, Malaysia; Islamic Azad University Damghan Branch, ISLAMIC REPUBLIC OF IRAN

## Abstract

Plant-based anticancer agents have the potential to stimulate the immune system to act against cancer cells. A standardized bioactive subfraction of the Malaysian herb, *Strobilanthes crispus* (L.) Blume (*S*. *crispus*) termed F3, demonstrates strong anticancer effects in both *in vitro* and *in vivo* models. The anticancer effects might be attributable to its immunomodulatory properties as *S*. *crispus* has been traditionally used to enhance the immune system. The current study examined whether F3 could stimulate anti-tumorigenic immunogenicity against 4T1 cells *in vitro* and in 4T1 cell-induced mammary carcinoma mouse model. We observed that F3 induced significant increase in MHC class I and class II molecules. CD4^+^, CD8^+^ and IL-2^+^ (*p*<0.05 for all) cells infiltration was also significantly increased in the breast tumor microenvironment of F3-treated mice compared with the tumors of untreated mice. The number of CD68^+^ macrophages was significantly lower in F3-treated mice. We conclude that the antitumor and antimetastatic effects of *S*. *crispus* involve strong infiltration of T cells in breast cancer potentially through increased tumor antigen presentation via MHC proteins, as well as reduction of infiltrating tumor-associated macrophages.

## Introduction

Globally, breast cancer is the most commonly diagnosed cancer, and is the leading cause of cancer-related deaths in women, in 2020 [1,2]. Human breast cancer-related deaths are mostly due to distant metastasis [3]. New cases of breast cancer are projected to increase multiple folds by 2025 without adequate public awareness initiative as well as early detection and provision of optimum patient care [4,5]. Immunotherapeutic strategies of breast cancer could affect tumor microenvironment via stimulation of specific immune cells to strengthen antitumor response [6] via a combination of innate and adaptive immunity.

More than 70% of tumor stroma in breast cancer is infiltrated by different subpopulations of lymphocytes particularly CD8^+^ T cells and CD4^+^ T helper (Th) cells which can detect tumor antigens in immunocompetent hosts to initiate killing of cancer cells [7,8]. Similarly, modern treatment approaches such as therapeutic monoclonal antibodies take advantage of the human immune system for destruction of breast cancer cells. Herceptin® (trastuzumab) is a standard drug against breast cancer which can interfere with human epidermal growth factor receptor 2 (HER2) function in tumor growth and metastasis [9,10]. Promising immunotherapeutic approaches include immune checkpoint inhibitors such as cytotoxic T lymphocyte-associated antigen 4 (CTLA4) antibodies and programmed cell death protein 1 (PD1). These have demonstrated antitumor effects and longer response in several clinical trials including metastatic and triple negative breast cancers (TNBCs) [11].

Immunomodulatory potentials of medicinal plants have been shown to mitigate growth and metastasis of many cancers including breast cancer [12,13]. As such, a number of medicinal plants are being evaluated as alternatives to synthetic anticancer drugs [14–16] such as tamoxifen and anastrozole which could cause undesirable effects and development of chemoresistance by breast cancer cells, especially in adjuvant tamoxifen therapy for breast cancer [17,18]. *Strobilanthes crispus* (L.) Blume (*S*. *crispus*) is a Malaysian herb locally known as ‘*Pecah kaca*’ or ‘*Jin batu*’ which is traditionally used to treat cancer [19,20] and to boost the immune system [21]. A standardized bioactive subfraction of *S*. *crispus*, termed F3, demonstrated strong anticancer effects in both *in vitro* and *in vivo* models [22–24]. F3 caused regression of N-methyl-N-nitrosourea (NMU)-induced mammary tumor in rats [24]. Recently, F3 was shown to inhibit migration and invasion of human MDA-MB-231 and murine 4T1 breast cancer cell lines as well as to suppress cancer progression in 4T1 mammary tumor model by disrupting the metastatic process. The anti-metastatic effect was accompanied by restoration of E-cadherin expression and downregulation of epithelial-mesenchymal transition (EMT) proteins [23]. These findings suggest the potential of F3 to help improve patient survival rate through prevention of metastasis. Evasion of immune destruction is another important characteristic of cancer [25] and EMT is one of the phenotypic changes that cancer cells undergo to escape immune surveillance [26].

In the current study, the ability of F3 to activate the immune system in the highly tumorigenic and invasive 4T1 cells and 4T1 cell-induced mammary carcinoma mouse model was investigated. 4T1 mouse mammary tumor model is suitable for assessment of metastasis due to its similarity to human breast cancer, particularly to determine immunotherapeutic response via infiltration of CD8^+^ and CD4^+^ T cells [27,28]. For instance, metastatic disease spreads to the lungs and liver in about 24% to 77% and 22% to 62% of women, respectively, compared to greater than 95% and 75%, respectively, in 4T1-induced mouse mammary carcinoma model. Bone metastasis is estimated at 70% in both human and murine 4T1 breast cancers. However, both cancers demonstrate lower occurrence of central nervous system involvement in metastasis [27,29,30]. In the current study, flow cytometry was performed to evaluate the expression of antigen presentation-related proteins (MHC class I and class II) in 4T1 cells treated with F3. Immunohistochemistry (IHC) analysis of immune cell populations (CD4, CD8 and CD68), cytokine (IL-2) and surface proteins (MHC class I and class II) were carried out to quantify their expression in mammary tumor tissues.

## Materials and methods

### Plant bioactive fractions

F3 was prepared by the Centre for Drug Research, Universiti Sains Malaysia (USM) from *S*. *crispus* leaves purchased from Agrodynamic Resources (Malaysia), located in Pulau Pinang, Malaysia. The plant was authenticated by the USM School of Biological Sciences that also deposited a voucher specimen of the plant (No. 11046). F3 was prepared from powdered freeze-dried leaves of *S*. *crispus* as previously described [24]. Briefly, fresh *S*. *crispus* leaves were freeze-dried and pulverized into powder form and sequentially extracted with hexane, dichloromethane and methanol in an ultrasonic cleaning bath (42 kHz, 185 W, 20 min) followed by an overnight soak. The extracts were filtered, and the solvent was evaporated in vacuo at a temperature below 35°C. The dichloromethane extract which was significantly cytotoxic to breast cancer cell lines, was further purified using dry vacuum liquid chromatographic technique on silica gel 60 (220 g). The fractions were then collected following step gradient elution with hexane: CHCl_3_-EtOAc-MeOH (2:3:0:0 to 0:0:9:1, v/v/v/v). F3 subfraction that was found to be most cytotoxic was further characterized. and bioactive compounds identified were lutein, β-sitosterol, stigmasterol, 13^1^-hydroxy-13^2^-oxo-pheophytin a, campesterol, pheophytin a, and 13^2^- hydroxy-pheophytin a [24].

### Breast cancer cell line

The 4T1 mouse breast cancer cell line (catalog no. CRL 2539) was directly purchased from American Type Culture Collection (Manassas, VA, USA) and immediately stored in the liquid nitrogen, upon arrival. Prior to cell culture, the frozen cells were placed in the -20°C freezer for 2 h and then briefly on ice before placing in a 37°C water bath for complete thawing. The cells were then transferred into a sterile centrifuge tube containing pre-warmed Roselle’s Park Memorial Institute (RPMI) medium at 37°C and centrifuged at 1,000 g for 5 mins. The cell pellet was resuspended in 5 ml pre-warmed RPMI medium and pipetted into 25 cm^2^ tissue culture flasks. The cells were observed under an inverted phase-contrast microscope for viability and morphology (cobblestone-like). The cells were maintained at 37°C in a humidified CO_2_ incubator (5% CO2) in RPMI medium, enhanced with 10% fetal bovine serum (FBS) and subcultured at 70–80% confluence. For experimental purpose, cells were subcultured in RPMI containing 2% FBS.

### Flow cytometric analysis

Flow cytometry was used to determine the expression of membrane-associated proteins (MHC class I and MHC class II) in 4T1 cell lines treated with F3 (50 μg/ml), dissolved in 0.1% dimethyl sulphoxide, for 24 h. The dose concentration of F3 was previously shown to inhibit 4T1 cells in vitro in a dose- and time-dependent manner [23]. The primary antibodies used were as follows: Anti-MHCI (Rabbit monoclonal, EPR1394Y; Abcam, Cambridge, UK); Anti-MHCII (Mouse monoclonal, MRC OX-6; Abcam); Rabbit IgG isotype control (EPR25A, Abcam); Mouse IgG1 isotype control (X092701, Dako Denmark A/S). Untreated cells and isotype controls were used as negative controls. Cellular expression of a specific protein was based on the detection of target antigen bound with fluorescent-labeled antibody at equilibrium. For a successful fluorescence activated cell sorting (FACS) of live cells using the flow cytometer, the calibration procedure, reagent preparations and dilution of antibodies were carefully performed to obtain optimum intensity of the fluorescence signal. Data for 10,000 live events were acquired for each sample using a FACS Calibur cytometer for analysis. The FACS results were analyzed using the FlowJo v.X.0.7 software (Tree Star Inc., Ashland, OR, USA).

### Experimental animals

Female Balb/c mice of 4 to 6 weeks old were purchased from the Animal Research and Service Centre, USM. The animals had free access to tap water and fed *ad libitum*, and were left to acclimatize for one week before the start of the experiment. This study was reviewed and approved by the USM Institutional Animal Care and Use Committee [USM/Animal Ethics Approval/2011/(69) (304)]. All experimental procedures were carried out according to the institutional relevant guidelines and regulations.

### Induction of mammary tumor and animal treatment

The 4T1 murine mammary carcinoma cells (0.1 ml equivalent of 10^5^ cells suspended in phosphate buffered saline) were inoculated into the right flank of the mammary fat pad of the experimental animals, based on the methods of Pulaski and Ostrand-Rosenberg [27], and Xanthopoulos et al. [31] with some modifications.The entire protocol of animal inoculation was performed in a sterile environment using biohazard safety cabinet class II (LabGard) NuAire, USA. The animals were properly restrained by grasping the loose skin at the back side of the neck using the thumb and index finger, and a 25-gauge needle was used for injection of 4T1 cells. Post inoculation, the mice were housed in individually ventilated cages (Tecniplast, Italy) in climate-controlled room with 14 h light-10 h dark cycle, 23 ± 2°C and 70 ± 5% of temperature and relative humidity, respectively. The animals were closely monitored daily for signs of distress and pain including inappetence, progressive body weight loss, rough hair coat, swollen abdomen, localized or general infection, discharges from external orifices and tumor ulceration. The attending veterinarian was consulted for advice and proper intervention when necessary. Palpable tumors developed within 2 weeks after induction. The tumor burden was monitored to not exceed 10% of the animal body weight. Moribund animals would be sacrificed early, but for the current study, none of the mice reached moribund stage and the experiment was concluded as planned.

As the tumor size progressed to 2 mm, the tumor-bearing animals were divided randomly into two groups (each n = 5) of untreated tumor-bearing mice (TM) and tumor-bearing mice treated with F3 (TM-F3) dissolved in corn oil. The sample size was determined based on the method of Pulaski and Ostrand-Rosenberg [27]. The TM-F3 group mice were orally administered with F3 (100 mg/kg/day) using dosing cannula for 30 days, as previously described and the selected dose has been reported to be non-toxic to normal mice [32]. The normal mice control group (NM, without tumor or treatment) was also included (n = 5). At the end of the experiment, all animals were humanely sacrificed, and the mammary glands and tumor nodules were harvested for IHC staining. Based on previous reports, anesthesia was first established using intraperitoneal injection of sodium pentobarbitone 40 mg/kg body weight [33,34]. The blood sample was then slowly withdrawn from the heart using 25G needle to prevent collapsing of the heart. Euthanasia was achieved by opening of the thoracic cavity to induce pneumothorax, and the animals were confirmed dead by the loss of heartbeat and respiration, response to painful stimuli and reflexes such as corneal, pinnae and pedal.

### Immunohistochemistry

IHC staining was performed on 3 μm sections from mammary glands or mammary tumor nodules to determine the expression of immune cell biomarkers, using anti-CD4 (rabbit monoclonal, ab237722; Abcam), anti-CD8 (rabbit monoclonal, ab209775; Abcam), anti-CD68 (mouse monoclonal, KP1; Abcam), anti-IL-2 (rabbit polyclonal, ab180780; Abcam) and surface proteins (MHC class I and class II) in NM, TM and TM-F3 groups. Tissue sections from both the positive and negative control groups were prepared concurrently for IHC staining with corresponding tissue sections of TM and TM-F3 groups. The tissue sections were incubated at 60°C for 10 mins and then deparaffinised in two changes of xylene for 5 mins each. This was followed by rehydration in two changes of absolute ethanol for 2 mins each and further rehydration in 95, 80 and 70% ethanol for 2 mins each before washing in dH_2_O. Subsequently, the tissue sections were immersed in Tris-EDTA (pH 9.0) buffer and placed in the decloaking chamber for boiling at 25 psi for 10 mins to induce antigen epitope retrieval. The sections were then blocked with peroxidase blocking solution for 5 min, and incubated with primary antibodies against CD4, CD8, CD68, IL2, MHCI or MHC II (Abcam, Cambridge, UK; 1:200 dilutions) for 1 h at room temperature. Following washing, the sections were incubated with Ultravision Quanto detection system HRP secondary antibody (Thermo Scientific, USA) for 10 min and with DAB-substrate chromogen complex for 3 min. The sections were counterstained with Harris hematoxylin (Sigma Aldrich) and mounted using cytoseal XYL^TM^ mounting medium (Thermo Scientific) for examination under light microscopy. Tissue sections were evaluated according to the modified Allred score and the number of positively stained cells was manually quantified as defined previously [35] with some modifications [23].

### Statistical analysis

For the *in vitro* study, statistical significance was calculated using Mann-Whitney test for comparison of the difference between F3-treated 4T1 cells and isotype control. Data were obtained from three separate experiments and the results were presented as mean fluorescence intensity (MFI) with interquartile range (IQR) in brackets. *In vivo* studies were analyzed using statistical package for social sciences (SPSS) Version 22. The results were presented as median with IQR in brackets. The non-parametric Kruskal-Wallis test was applied for determination of significant differences between TM and TM-F3 groups. Mann-Whitney test was used as the post hoc test for comparison between NM or TM and TM-F3 groups. In this study, *p*<0.05 was considered statistically significant.

## Results

### Expression of MHC class I and II in 4T1 cells

**Fig 1:**
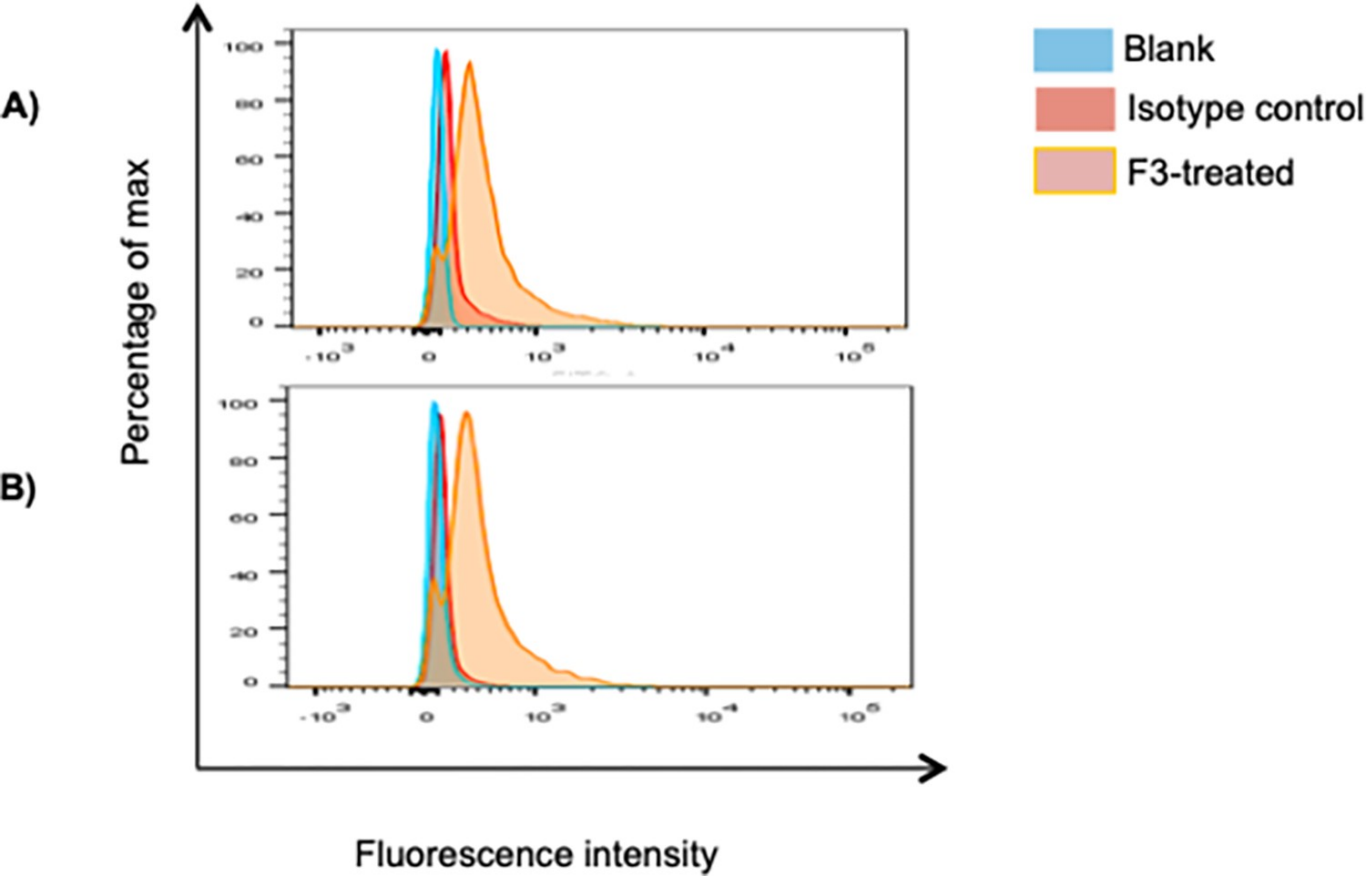
Expression of (A) MHC class I and (B) MHC class II on 4T1 cells treated with F3. The FACS results were analyzed using the FlowJo v.X.0.7 software (Tree Star Inc., Ashland, OR, USA).

The expression of membrane-associated MHC class I and class II proteins in 4T1 cell line (Fig 1) was determined by flow cytometry, using the vehicle (untreated) and isotype controls as negative controls. Treatment of 4T1 cells with F3 for 24h significantly increased the expression of MHC class I (*p* = 0.008) and MHC class II (*p* = 0.034) surface proteins, when compared with the untreated control cultures (Table 1). Comparison with the isotype control also yielded significant expression for both proteins (MHC class I, *p* = 0.020) and (MHC class II, *p* = 0.021).

**Table 1:** Effects of F3 on the expression of MHC class I and class II in 4T1 cells.

Cell surface molecules	Blank (MFI)	Isotype Control (MFI)	F3-treated (MFI)
MHC class I	63.0 (5)	142.0 (17.0)	364.0 (70.0) **^,^ ^#^
MHCII class II	86.0 (63)	116.0 (5.0)	354.0 (29.0) *^,^ ^#^

*p<0.05 and **p<0.01 in comparison with vehicle control

^#^*p<*0.05 in comparison with isotype controls.

### Expression of MHC class I and class II in 4T1-induced mammary tumors

**Fig 2:**
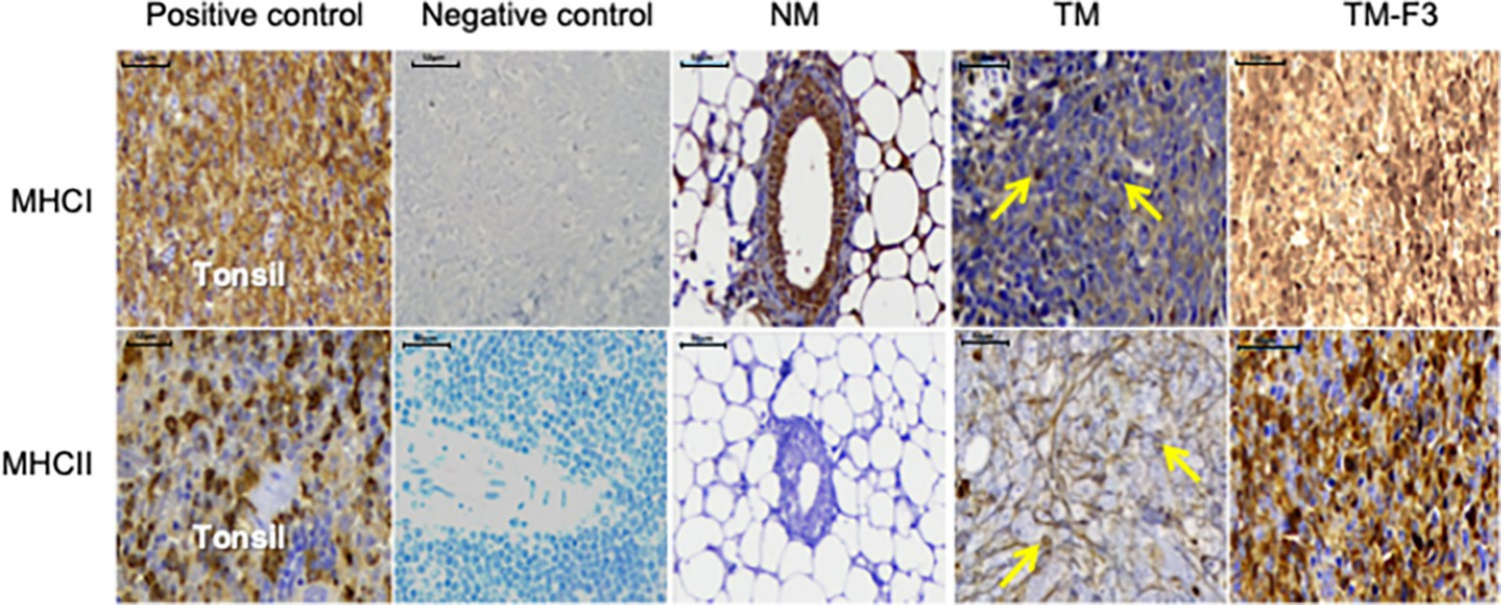
Representative photomicrographs for expression of MHC class I and class II molecules in untreated and treated tumors. Arrows indicate positively stained mammary tumor cells. Images were captured at X400 magnification. The scale bar corresponds to 50 μm.

**Fig 3:**
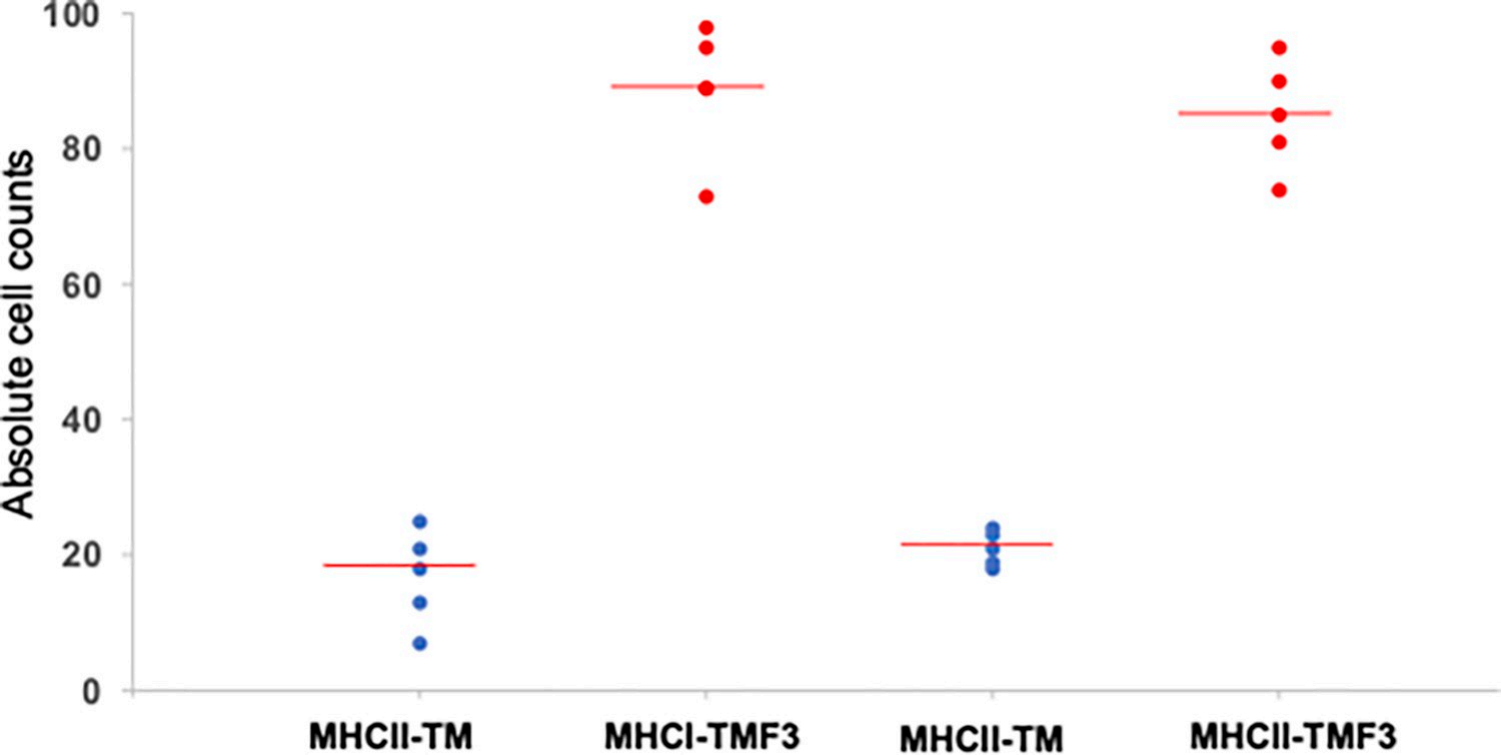
Absolute counts for expression of MHC class I and class II molecules in treated and untreated tumors. *P* values were calculated using Mann-Whitney test for comparison of absolute cell counts between untreated (TM) and treated (TM-F3) tumor-bearing mice.

The expression of MHC class molecules in mouse mammary glands and 4T1-induced mammary tumors was determined by IHC. Representative photomicrographs of these markers in NM, TM and TM-F3 groups are shown in Fig 2. There was increased expression of MHC class I detection in breast tissues of NM or TM-F3 compared to the TM group. F3 induced significant increase in MHC class II molecule in treated breast cancer tumors compared with the untreated tumors of the TM group. However, normal mouse breast tissue sections showed no expression of MHC class II molecule. Assessment of the IHC staining was performed using absolute count (*i*.*e*. the average of the positively stained cells counted in each of the four randomly selected fields), proportion score (percentage of viable cells showing staining), intensity score (measured using a scale of 0–3, where 0 = negative staining, whereas 1 = weak, 2 = moderate and 3 = strong positive staining with reference to the corresponding control slide) and overall score (determined by adding the intensity score and the score obtained from the number of positively stained cells). IHC assessment was conducted independently for each marker to indicate significant upregulation or downregulation of MHC class I or MHC class II molecule in the mammary glands or mammary tumor tissues of the experimental animals (Table 2). Fig 3 shows that the mean absolute cell counts for MHC class I and class II expression are significantly higher following treatment of the tumors with F3.

**Table 2:** Effects of F3 on the expression of MHC class I and class II in TM-F3 when compared with the TM group.

Immune markers	Statistical Parameters	TM (n = 5)	TM-F3 (n = 5)	*p-*value
MHC class I	Proportion score	2.0 (1.0)	4.0 (1.0)	0.049
	Intensity score	2.0 (1.0)	3.0 (1.0)	0.007
	Overall score	4.0 (2.0)	7.0 (1.0)	0.006
MHC class II	Proportion score	1.0 (1.0)	3.0 (1.0)	0.005
	Intensity score	1.0 (1.0)	2.0 (1.0)	0.016
	Overall score	2.0 (1.0)	6.0 (2.0)	0.004

*P* values were calculated using Mann-Whitney test for comparison between untreated (TM) and F3-treated (TM-F3) groups. Results are presented as median values with IQR in brackets, and *p*<0.05 is considered statistically significant.

### Infiltrating T cells and tumor-associated macrophages at tumor sites

**Fig 4:**
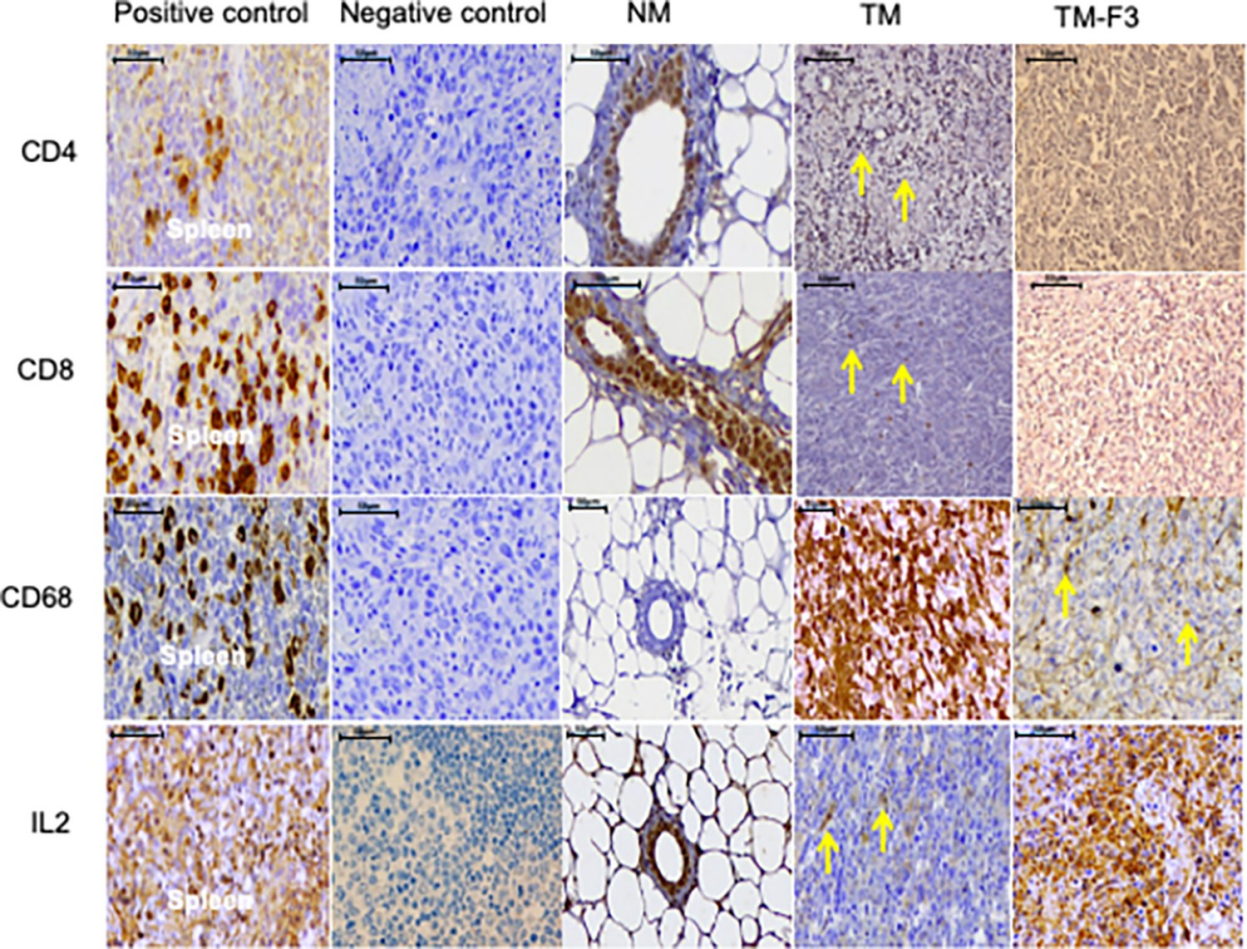
Representative photomicrographs showing expression of CD4, CD8, CD68 and IL-2 in treated and untreated tumors. Arrows indicate positively stained lymphocytes (CD4 & CD8), macrophages (CD68) and mammary tumor cells (IL2). Images were captured at X400 magnification. The scale bar corresponds to 50 μm.

**Fig 5:**
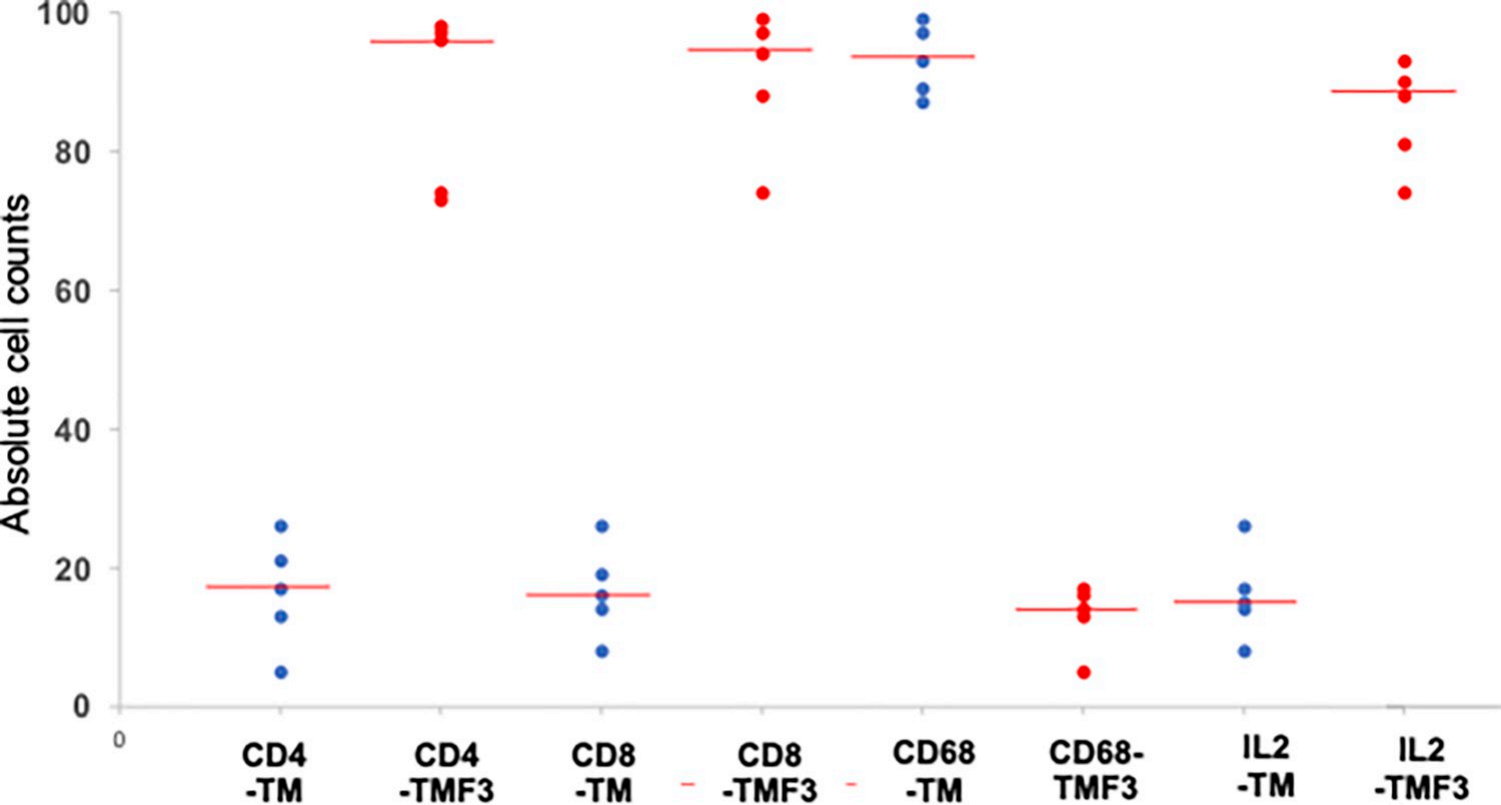
Absolute counts for expression of CD4, CD8, CD68 and IL-2 molecules in treated and untreated tumors. *P* values were calculated using Mann-Whitney test for comparison of absolute cell counts between untreated (TM) and treated (TM-F3) tumor-bearing mice.

F3 treatment increased the expression of MHC class molecules and this can potentially increase the presentation of tumor-associated antigens by MHC proteins. Hence, we subsequently investigated whether F3 treatment also increased the infiltration of T cells with decreased presence of tumor-associated macrophages (TAMs) as defined by CD68 positivity (Fig 4). As shown in Fig 5 and Table 3, statistical parameters determined (absolute count, proportion, intensity and overall scores) indicated significant increase (*p*<0.05) in the number of CD4^+^ (T helper cells), CD8^+^ (cytotoxic T cells) and IL-2^+^ (activated T cells) in mammary tumor tissues of TM-F3 when compared to the TM group. NM mammary tissue sections showed no expression for CD68 (TAMs); however, the number of CD68^+^ cells was significantly reduced in TM-F3 compared to the TM group (*p*<0.05). Significant differences in all these populations of cells between TM-F3 versus TM group were observed for all four parameters investigated *i*.*e*. absolute cell count, proportion score, intensity score, and overall score (Fig 5, Table 3).

**Table 3:** Effects of F3 on the expression of immune markers in TM and TM-F3.

Immune markers	Statistical parameters	TM (n = 5)	TM-F3 (n = 5)	*p-*value
CD4	Proportion score	1.0 (1.0)	4.0 (1.0)	0.006
	Intensity score	1.0 (1.0)	3.0 (1.0)	0.020
	Overall score	2.0 (2.0)	6.0 (1.0)	0.006
CD8	Proportion score	1.0 (1.0)	4.0 (1.0)	0.005
	Intensity score	2.0 (1.0)	3.0 (1.0)	0.014
	Overall score	3.0 (2.0)	7.0 (1.0)	0.006
CD68	Proportion score	4.0 (1.0)	1.0 (1.0)	0.006
	Intensity score	3.0 (1.0)	2.0 (1.0)	0.018
	Overall score	7.0 (1.0)	3.0 (2.0)	0.006
IL-2	Proportion score	1.0 (1.0)	4.0 (1.0)	0.005
	Intensity score	1.0 (1.0)	2.0 (1.0)	0.014
	Overall score	2.0 (1.0)	6.0 (2.0)	0.007

*P* values were calculated using Mann-Whitney test for comparison between untreated (TM) and F3-treated (TM-F3) groups. Results are presented as median values with IQR in brackets, and p<0.05 is considered statistically significant.

## Discussion and conclusion

The search for potent plant-based immunomodulatory anticancer agents has resulted in the discovery of many metabolites that stimulate the immune system against breast cancer [36,37]. The immune-potentiating effects of F3 demonstrated in this study corroborated with our previous findings in which F3 administration significantly increased the white blood cell count in NMU-induced rat mammary carcinoma model [24]. A closely related hematological indices were obtained between normal mice supplemented with F3 (NM-F3) and normal mice (NM) control group (S1 Table). Similarly, rats induced to have mammary tumor by NMU and treated with F3 had increased infiltrating T cell counts while the tumor cells displayed increased MHC class II expression [22]. A comparable pattern of immune mechanism was observed in the current study with significant increase in the number of infiltrating T cells (CD4^+^, CD8^+^ or IL-2^+^) and associated biomarkers (MHC class I and class II) in the TM-F3 group compared with the untreated TM group which also had higher number of CD68^+^ TAMs.

F3-induced activation of immune system conforms to the principle of immunotherapeutic strategies (e.g. trastuzumab) which take advantage of the immune system to destroy breast cancer cells [17,38] via tumor-infiltrating cells consisting of innate (natural killer cells and dendritic cells) and adaptive (CD4^+^ and CD8^+^ T cells) immune components [8,39]. As observed in this study, high density lymphocytic infiltration, particularly CD8^+^ cells, can stimulate T cell-mediated cytotoxicity which has been associated with favorable outcome in different cancers including breast cancer, and infiltration deeply into the tumor core by T cells is essential for effective antitumor immunity, overall survival and prevention of tumor recurrence [7,8]. On the other hand, loss of T cell immune response can lead to tumor metastasis even after long periods of dormancy [40]. Similarly, the increased expression of CD4^+^ cells by the F3 is suggestive of its role in antitumor response. It was found that neoadjuvant chemotherapy that stimulates high density CD4^+^ cell infiltration correlates with tumor regression in breast cancer [41]. Also, F3 could possibly induce Th1 activity of CD4^+^ T cells to stimulate IL-12, which in turn releases IL-2 (significantly expressed in TM-F3) and interferon-γ to enhance cell-mediated antitumor effects. Immune protective effects of F3 are perhaps resulted from the actions of multiple bioactive components present in F3, especially lutein and β-sitosterol which have been recognized to exert different biological effects on the immune system [42,43].

F3 caused increased production of IL-2 by the Th1 subset that could induce activation and movement of CD4^+^ and CD8^+^ T cells towards tumor microenvironment for destruction of breast cancer cells. IL-2 is a T cell-activating cytokine and is used as an immunotherapeutic strategy to stimulate T cell-dependent immune response in cancer [44]. Thus, elevated IL-2 expression due to treatment with F3 could perhaps induce greater expansion of cytotoxic T cells to inhibit breast cancer growth. Our previous study [22] evaluated the effects of F3 on the levels of 34 serum cytokines using the cytokine antibody array. We showed that F3 administration decreased the level of chemokine ligand 2, indicating reduced TAM infiltration. Additionally, the level of interferon gamma was increased, in line with enhanced T cell infiltration. F3 did not affect the serum levels of other chemokines or interleukins. Besides, the increased infiltration of essential T cell population, high density expression of MHC class I and MHC class II molecules by breast cancer cells in both the *in vitro* and *in vivo* studies observed in the current study, have further established the immunoprotective role of F3. F3-mediated upregulation of MHC class molecules indicates blockade of tumor evasion of the immunosurveillance and subsequent promotion of antigen presentation by MHC class I and MHC class II proteins required for tumor antigen presentation to the CD8^+^ and CD4^+^ T cells, respectively, for tumor elimination [45,46]. Moreover, it has been reported that improved expression of MHC class II-mediated antigen presentation signaling mechanism correlated with good prognosis in TNB cancer, and this could trigger antitumor response that decreased rate of recurrence and enhanced overall survival [47]. Likewise, an immune signature comprising of high MHC class I and T cell density has been predicted as prognostic tool of therapeutic response in colorectal liver metastasis [48]. Our current findings further support the previously reported downregulation of EMT markers and improvement of E-cadherin expression in F3-treated tumors [23].

Moreover, breast cancer immune signature where high expression of CD68 and CD4 along with low expression of CD8 correlates with reduced survival and higher relative risk for metastasis [49]. Others have also correlated high grade TAMs with poor prognosis and reduced survival rate [50,51]. Administration of F3 to tumor-bearing mice has downregulated the number of CD68^+^ TAMs. TAMs are forms of activated macrophages that promote tumor progression by inducing angiogenesis, invasion and metastasis [52–54]. TAMs express a number of macrophage-specific markers including CD68 [53] and should therefore be evaluated in the future as part of broad assessment of CD68 immune response by F3. In tumor immunity, studies on breast cancer have demonstrated that high expression of T helper CD4^+^ and CD8^+^ cytotoxic T cells were associated with good prognosis and overall survival [7,8]. Furthermore, immunomodulatory potentials of F3 could possibly inhibit tumor cell evasion of the immune system and subsequent metastasis, as a result of increased infiltration of T helper CD4^+^ and CD8^+^ cytotoxic T cells. Thus, this could trigger strong immune surveillance to destroy breast cancer cells. Immune evasion is one of the hallmarks of cancer and thus immune competence is critical to immunotherapeutic strategies in breast cancer [25,55]. Similarly, chemotherapeutic approaches which take benefit of tumor-infiltrating immune cells possess potentials in preventing breast cancer metastasis [56].

Overall, the findings in this study support the immune-activating properties of F3 against breast cancer cells. This is achieved via reduced TAMs but increased infiltration of T cells (Th cells and cytotoxic T cells) at the tumor site, potentially due to increased tumor antigen presentation via increased MHC class I and MHC class II molecules expression.

## Supporting information

S1 Table Full blood count indices in normal mice and normal mice treated with F3. The FBC values were calculated using Mann-Whitney test for categorical data between normal mice and normal mice treated with F3. Results are presented as median values with IQR in brackets. There was no statistical difference as compared to NM.
(DOCX)
