# Supplementary material for: Strobilanthes crispus elicits anti-tumor immunogenicity in in vitro and in vivo metastatic breast carcinoma
Source: PLoS One. 2022 Aug 16;17(8):e0271203. doi: 10.1371/journal.pone.0271203 (PMC9380931; doi:10.1371/journal.pone.0271203)
Supplement: S1 Table — The FBC values were calculated using Mann-Whitney test for categorical data between normal mice and normal mice treated with F3. Results are presented as median values with IQR in brackets. There was no statistical difference as compared to NM. (DOCX) [file pone.0271203.s001.docx]

**S1 Table. Full blood count indices in normal mice and normal mice treated with F3.**

| FBC indices | Normal mice  (n = 5) | F3-supplemented  (n = 5) | |
| --- | --- | --- | --- |
| Hemoglobin (g/dl) | 159.0 (8.00) | | 158.0 (7.0) |
| Packed cell volume (%) | 0.55 (0.03) | | 0.56 (0.03) |
| Red blood cells (x 10^12/L) | 9.6 (0.50) | | 9.8 (0.60) |
| Mean corpuscular volume (fl) | 57.0 (1.00) | | 57.0 (1.00) |
| Mean corpuscular haemoglobin (pg) | 16.0 (1.00) | | 16.0 (1.00) |
| Mean corpuscular haemoglobin concentration (g/L) | 280.0 (10.00) | | 280.0 (10.00) |
| Red cell distribution width (%) | 16.7 (1.10) | | 16.7 (0.70) |
| White blood cells (x 10^9/L) | 1.1 (0.50) | | 1.6 (2.30) |
| Polymorphs (%) | 29.0 (13.00) | | 29.0 (15.00) |
| Lymphocytes (%) | 71.0 (15.00) | | 63.0 (15.00) |
| Monocytes (%) | 2.0 (3.00) | | 3.0 (4.00) |
| Eosinophils (%) | 0.0 (1.00) | | 1.0 (1.00) |
| Basophils (%) | 0.0 (0.00) | | 0.0 (0.00) |
| Platelets (x 10^9/L) | 540.0 (385.00) | | 631.0 (192.00) |
